# Effects of caffeine and blue-enriched light on spare visual attention during simulated space teleoperation

**DOI:** 10.1038/s41526-023-00299-8

**Published:** 2023-12-19

**Authors:** Andrew M. Liu, Raquel C. Galvan-Garza, Erin E. Flynn-Evans, Melanie Rueger, Alan Natapoff, Steven W. Lockley, Charles M. Oman

**Affiliations:** 1 Human Systems Laboratory, Massachusetts Institute of Technology, Cambridge, MA 02139 USA; 2 Division of Sleep and Circadian Disorders, Departments of Medicine and Neurology, Brigham and Women’s Hospital, Boston, MA 02115 USA; 3 Division of Sleep Medicine, Harvard Medical School, Boston, MA 02115 USA; 4 Fatigue Countermeasures Group, Human Systems Integration Division, NASA Ames Research Center, Moffett Field, California, CA 94035 USA

**Keywords:** Medical research, Translational research

## Abstract

Safe and successful operation of the International Space Station robotic arm is a complex task requiring difficult bimanual hand coordination and spatial reasoning skills, adherence to operating procedures and rules, and systems knowledge. These task attributes are all potentially affected by chronic sleep loss and circadian misalignment. In a randomized, placebo-controlled, cross-over trial examining the impact of regularly timed low-dose caffeine (0.3 mg kg^−1^ h^−1^) and moderate illuminance blue-enriched white light (~90 lux, ~88 melEDI lux, 6300 K), 16 participants performed 3 types of realistic robotic arm tasks using a high-fidelity desktop simulator overnight. Our goal was to determine how these countermeasures, separately and combined, impacted telerobotic task performance and the ability to allocate attention to an unrelated secondary visual task. We found that all participants maintained a similar level of robotic task performance throughout the primary task but the application of caffeine separately and with blue-enriched light significantly decreased response time to a secondary visual task by −9% to −13%, whereas blue-enriched light alone changed average response times between −4% and +2%. We conclude that, for sleep-restricted individuals, caffeine improved their ability to divide their visual attention, while the effect of blue-enriched light alone was limited. Light and caffeine together was most effective. Use of these countermeasures should improve the margin of safety if astronauts perform familiar tasks under degraded conditions or novel tasks where task workload is increased.

## Introduction

The companion paper Flynn-Evans et al.^1^ demonstrated that caffeine and blue-enriched light countermeasures significantly reduced the impairment in short-term memory and reaction time, as measured by a cognitive performance test battery, and improved EEG-derived objective correlates of alertness. We also examined the effects of these countermeasures on performance and workload measures while completing a series of complex robotics tasks. Little is known about the effects of sleep restriction and fatigue on the performance of complex tasks requiring a combination of multiple interconnected steps, well-practiced sensorimotor and spatial skills, adherence to procedures and rules, and decision-making based on systems knowledge. Our hypothesis was that the countermeasure effects on cognitive and robotics test battery metrics would correlate, particularly those measures involving visual attention, vigilance, and higher-level reasoning.

**Fig. 1:**
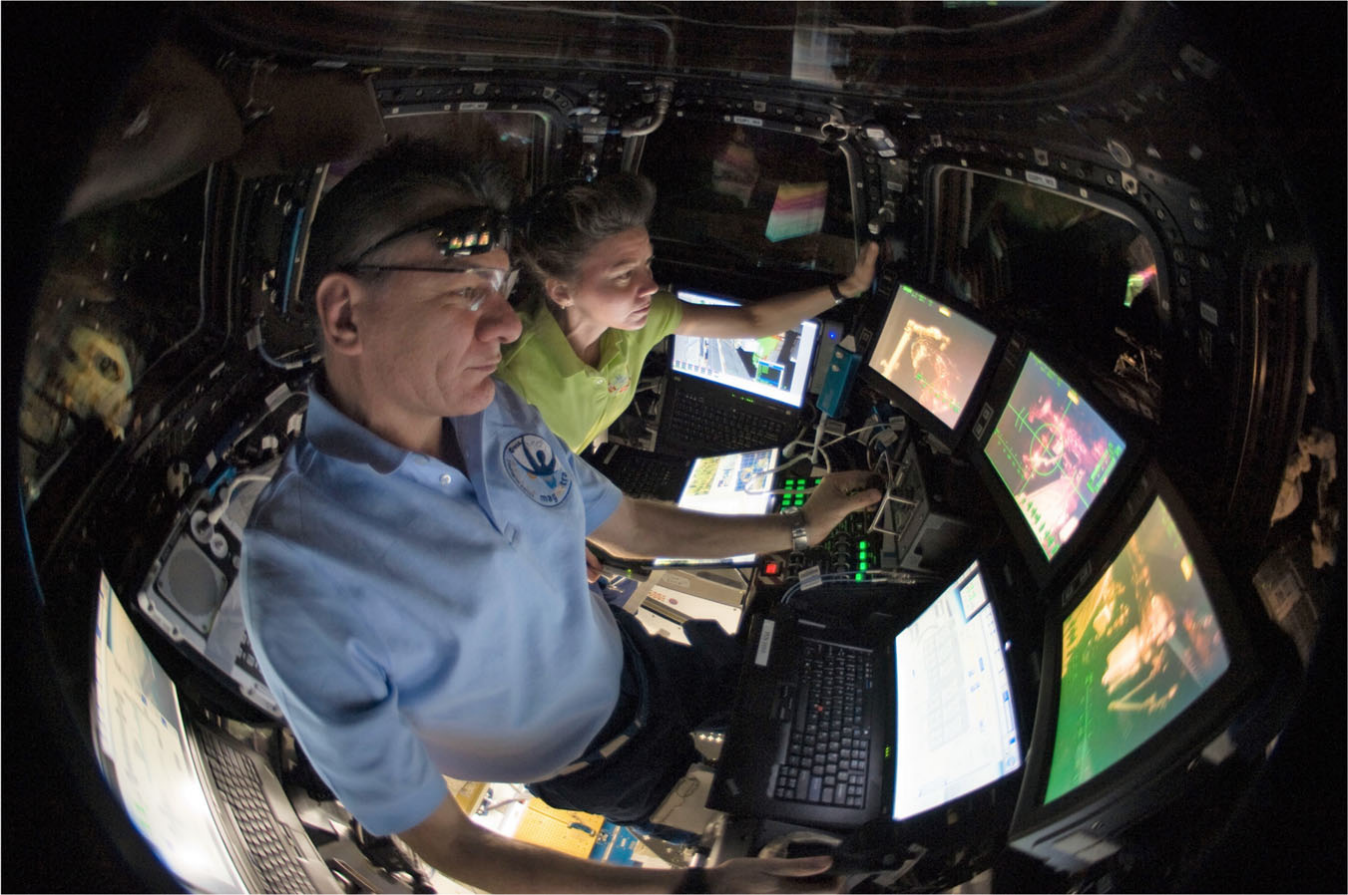
Robotic Workstation installed in the ISS Cupola. Astronauts Paolo Nespoli and Cady Coleman receive visual feedback from three or more displays and control robot arm movements with two hand controllers. (Photo: NASA. Images are published with permission from Paolo Nespoli and Cady Coleman).

What makes space teleoperation difficult? There are many challenging aspects involving many areas of perception, cognition, and action. At the perceptual level, operators must remain vigilant and continuously integrate separate two-dimensional camera views into a three-dimensional perception of the arm’s orientation to maintain safe clearance from structures (Fig. 1). Operators are taught to recognize specific configurations and employ mental checklists and rules for clearance and strategies for camera selection and trajectory planning. Although arm motions can be preprogrammed and performed automatically, most operations require some intervals of manual control. At the motor skill level, operators must learn to decompose the desired motion of the robotic arm into coordinated bi-manual rotational and translational hand controller inputs. Determining the direction of arm motion produced by a specified hand controller input is not always intuitive since it depends on the camera view and selected control mode, both of which may need to be changed several times. Generically, there are three classes of space robotics tasks: “capturing” which involves using the hand controllers to grapple an object with the end of the arm, “positioning” which requires moving the object to a new location, and “monitoring” when the robotic arm is moving entirely automatically. Successful completion of these tasks relies on fast and accurate spatial processing, sustained attention, memory recall and executive function, all which can be adversely affected by sleep and circadian factors. To become a certified operator, astronauts complete hundreds of hours of training and practice to master robotic arm operations. The goal is to make the bi-manual control automatic, to teach operators rules and strategies appropriate for the situation, to reduce demands for high level reasoning and, thereby, reducing mental workload.

Research has shown that adding a lower priority “secondary” task, which competes for processing resources with the primary task provides a proxy measure of the spare processing capacity and mental workload, as explained by multiple resource theory^2^. Task “mental workload” is typically distributed over several cognitive subsystems, each with a limited processing capacity. For workload assessment during space robotic tasks, adding a secondary visual attention task, such as responding to intermittent visual stimuli, provides a useful workload proxy since visual attention is required for several subtasks, including tracking the robot arm position and checking robot arm clearance from any structures. Other studies have shown that visual secondary tasks are sensitive to manipulations of operator’s time awake in simulated space robotics tasks^3^ and automobile driving^4,5^. In some cases, probing spare attention capacity through secondary task performance may provide the only sensitive and reliable measure on the effects of sleep restriction. Kahol et al. found that sleep-deprived helicopter pilots were able to control their vehicle within acceptable limits, but made cognitive and judgement errors such as flying on the wrong course or miscalculating the location to initiate a turn^6^. Similarly laparoscopic surgical residents had more difficulty with cognitive skills, such as planning or memory recall after an overnight shift than before their shift, although their motor skills were rated to be similar^7^.

While all robotic arm operations aboard the Space Shuttle and International Space Station (ISS) to date have been completed, operational errors with potentially serious consequences (e.g., improper entry of parameters for automatic arm operation; failure to identify potential clearance violations) have occurred to which operator fatigue has been cited as a contributing factor^8^. Some of these errors were caught by ground personnel following the operations who intervened before the situation became critical, but astronauts on future exploration missions beyond low-earth orbit will have to operate more independently. Thus, preventative sleepiness countermeasures are necessary to reduce the risk of task failure and improve crew safety.

The goal of this study was to identify the effects of caffeine and blue-enriched white light countermeasures on spare visual attention and robotics task performance in participants who had experienced sleep restriction and changes in their sleep-wake schedule. Our experiment design and analysis allowed us to separate countermeasure condition effects from individual participant, learning and time-on-task effects. We knew that primary robotics task performance would vary considerably among participants, making countermeasure effects difficult to detect, but hypothesized that use of caffeine and/or blue-enriched white light would have detectable effects on proxy measures of mental workload and visual attention sharing. Furthermore, we anticipated that the relative magnitude of the countermeasure effects would be similar to those detected in concurrent cognitive and physiological tests^1^.

## Results

### Experiment Conditions

**Fig. 2:**
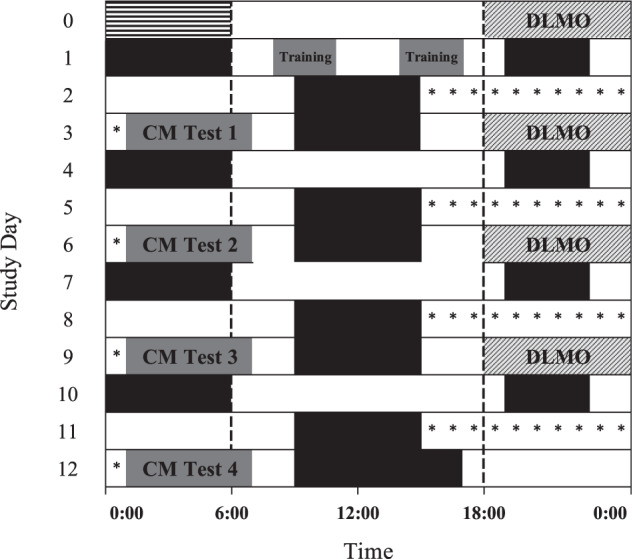
Protocol schedule. Participants completed the tasks of the robotics test battery in the order shown above over a 1-h period (dark gray bars). They repeated the robotics test battery four times within the 6-h test countermeasure session. The same 12 Capture Task trials were performed in each repetition of the robotics test battery but in a different order. Each of the Position Task and Monitor Task trials was unique.

After maintaining a 6-h sleep schedule for the prior week, 16 healthy participants were admitted into a time-cue-free sleep laboratory for a 13-day inpatient study. They maintained 6 h of sleep per day but were slam shifted with a 9-h sleep delay four times as shown in Fig. 2. A test session was scheduled before each shifted sleep opportunity, and participants were therefore awake for 16 h by the end of each test session. The first test session on Day 4 was performed while exposed to standard ~90 lux (4700 K) white light and placebo, while the last three sessions were performed under one of three randomized countermeasure conditions: standard white light and hourly caffeine (0.3 mg kg^−1^ h^−1^), ~90 lux blue-enriched white light (6300 K) and placebo, or 90 lux blue-enriched white light and hourly. The order of countermeasure conditions was balanced across participants. Within each session, participants completed a 1-h robotics test battery followed by a 30-min cognitive test battery and repeated both four times for a total session duration of 6 h. Technical problems during Subject 14’s participation led to the loss of data for the first three blocks of the session using the blue-enriched white light and placebo condition.

The robotics test battery included capturing tasks, positioning tasks and monitoring tasks as described earlier. Robotics performance was measured by task completion time, frequency of adverse events and procedural errors, and metrics characterizing hand controller movements. Participants concurrently performed a secondary visual stimulus-response task during each robotic task with the stimulus reaction time as a proxy for their mental workload during the task. When the task was completed, the secondary task was also stopped, even if the task was completed before the allotted trial time. The cognitive test battery included the Visual Analogue Scale (VAS), the Karolinska Sleepiness Scale (KSS), the Digit Symbol Substitution Task (DSST), the 10-min visual version of the Psychomotor Vigilance Test (PVT), and the Karolinska Drowsiness Test (KDT). Most data were analyzed via mixed hierarchical regression. Additional details are available in the companion paper, Flynn-Evans et al.^1^.

### Secondary visual attention task performance

**Fig. 3:**
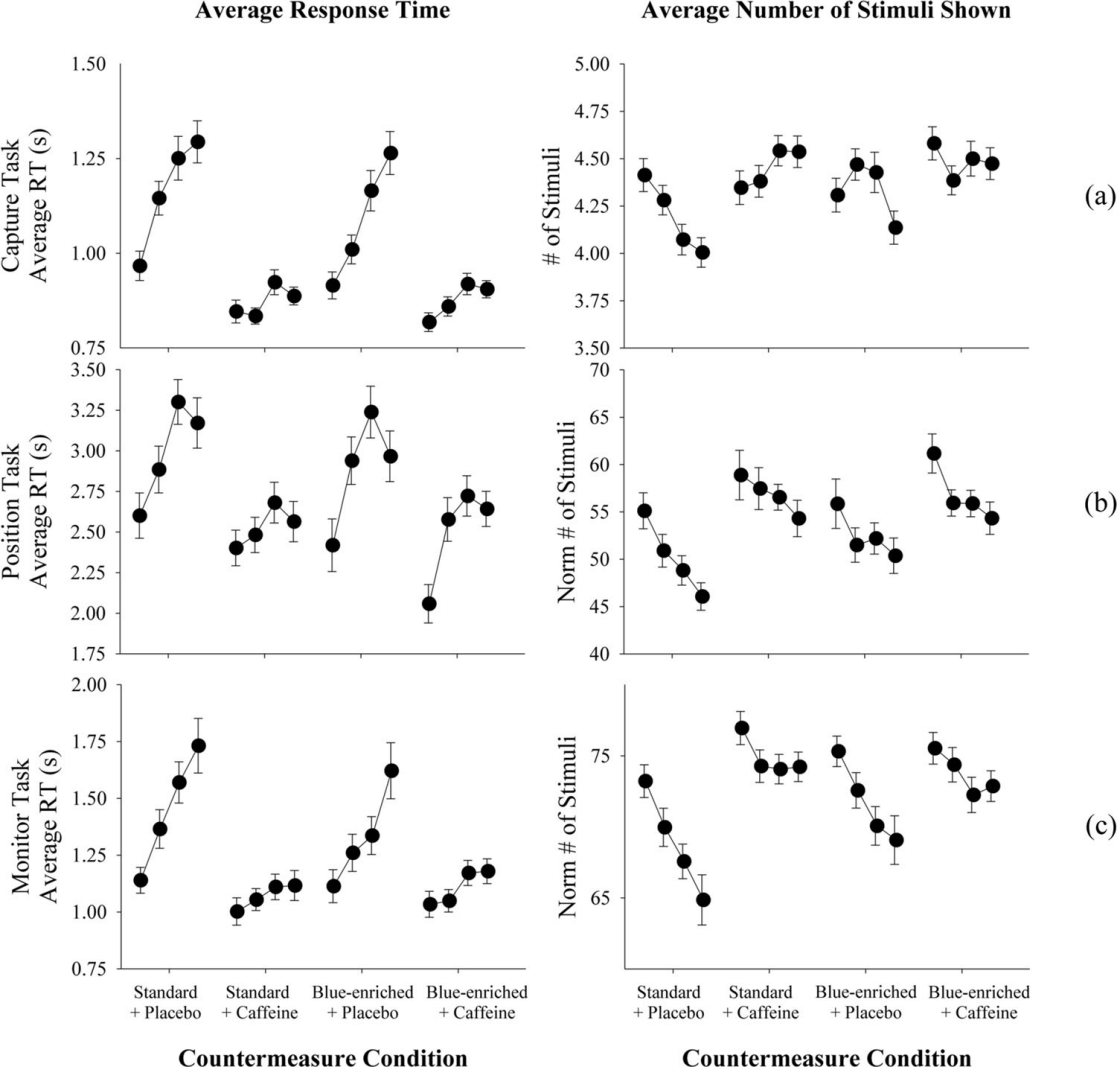
Secondary Task performance metrics. Each row represents one task. (**a**) Capture Task, (**b**) Position Task, and (**c**) Monitor Task. For each countermeasure condition, the robotics test battery was repeated four times. The left column shows the average response time to stimuli that were detected, and the right column shows the average number of stimuli presented per trial. Error bars represent the standard deviation. In general, performance was improved when caffeine was used (Standard 4100 K light + Caffeine and Blue-enriched 6500 K light + Caffeine conditions) compared to when it was not used (Standard 4100 K light + Placebo and Blue-enriched 6500 K light + Placebo conditions) with no degradation in performance over each of the four task repetitions.

As shown in the left panel of Fig. 3, the average secondary task response times were faster in the two caffeine countermeasure conditions compared to the no-countermeasure and the blue-enriched light-only countermeasure condition. Our mixed hierarchical regression analysis of countermeasure effects (see Methods) showed that for all task types, the average response times during the two caffeine conditions were significantly faster than response time averaged across all countermeasure conditions (*p* < 0.005 for both cases). The average response times of the remaining two treatment conditions were significantly longer than the overall average (*p* < 0.005 for both cases). The response time improvement due to the caffeine countermeasure (compared to the no-countermeasure condition) was estimated to be a 9–13% improvement. For blue-enriched white light, the estimated effects on average response time ranged from a 4% improvement in the Capture Task, no change in the Position Task to a 2% decline in the Monitor Task.

The analysis also showed that within each 6-h test session, the average secondary task response time increased with each repetition of the 1-h robotics test battery (*p* < 0.001), possibly due to time-on-task effects, as shown in Fig. 3, left. The time-on-task effect was estimated to be roughly half of the effect of caffeine. Average response time also decreased significantly with each successive 6-h test session for the Capture Task (*p* < 0.001) and the Monitor Task (*p* = 0.009), possibly due to learning effects, although this effect was 3–4 times smaller than the effect of caffeine.

If the participant did not respond to the secondary stimulus within 10 s, no response time was recorded. Therefore, the number of secondary stimuli presented during the test period is arguably another correlate of workload level since it accounts the metric’s value accounts for both missed stimuli and response time. When compared to the no-countermeasure condition, the average number of presented stimuli was significantly higher in the two caffeine countermeasure conditions whereas the blue-light-only countermeasure did not produce a difference (Fig. 3, right). During the Capture Task, the two countermeasure conditions that included caffeine presented a significantly greater number of secondary stimuli (both conditions, *p* < 0.01). There was also a significant decline in the number of stimuli presented across the four repetitions of the robotics test battery within a 6-h test session (*p* < 0.001). To analyze the average number of stimuli presented in a Position Task trial, we normalized the number of displayed stimuli over a 10-min period because the task completion time varied over several minutes across participants and trials. The effect of caffeine was highly significant (both conditions, *p* < 0.001). The effect of repetition of the robotics test battery was not evaluated since the tasks were different in each repetition. For the Monitor Task, the caffeine-only condition was highly significant (both conditions, *p* < 0.02) and an effect of repetition of the robotics test battery was also significant (*p* < 0.001).

### Primary robotics task performance

We quantified primary robotics task performance by the frequency of adverse events that could result in damage to the robotic arm or payload (e.g., collisions or failed grapple attempts), the frequency of arm control movements that violate procedural rules (e.g., moving the arm too close to another object or rotating a joint to its limit of motion), task completion time, and measures that characterize the smoothness and bimanual coordination of the participant’s control inputs. These are similar to the performance metrics used in astronaut training and other research using NASA robotics simulations^9^. Primary task performance metrics showed sufficient variability that no countermeasure effects were significant but several interesting trends in the mean effects are discussed below.

Table 1 compares the frequency of adverse events and procedural rule violations for the Capture and Position Tasks across the four conditions. The highest proportion of adverse events and procedural rule violations occurred in the no-countermeasure condition. In comparison, participants performed better in the three countermeasure conditions but with very little difference among them. Considering that the no-countermeasure condition was always performed first, this effect could simply be a learning effect.

**Table 1:** Rows 1–2: Proportion of trials involving a collision or failed grapple during the Capture and Position Tasks.Rows 3–4: Position Task trials involving at least one Clearance or Joint Limit error.

	None	Caffeine	Blue-enriched	Both
Capture: Failed trials	3.5%	2.5%	2.4%	1.9%
Position: Failed trials	21.3%	10.3%	16.1%	15.9%
Position: Clearance Error	74%	66%	65%	66%
Position: Joint Limit Error	27%	22%	25%	26%

Primary Task Performance Metrics.

Rows 3–4: Position Task trials involving at least one Clearance or Joint Limit error.

**Fig. 4:**
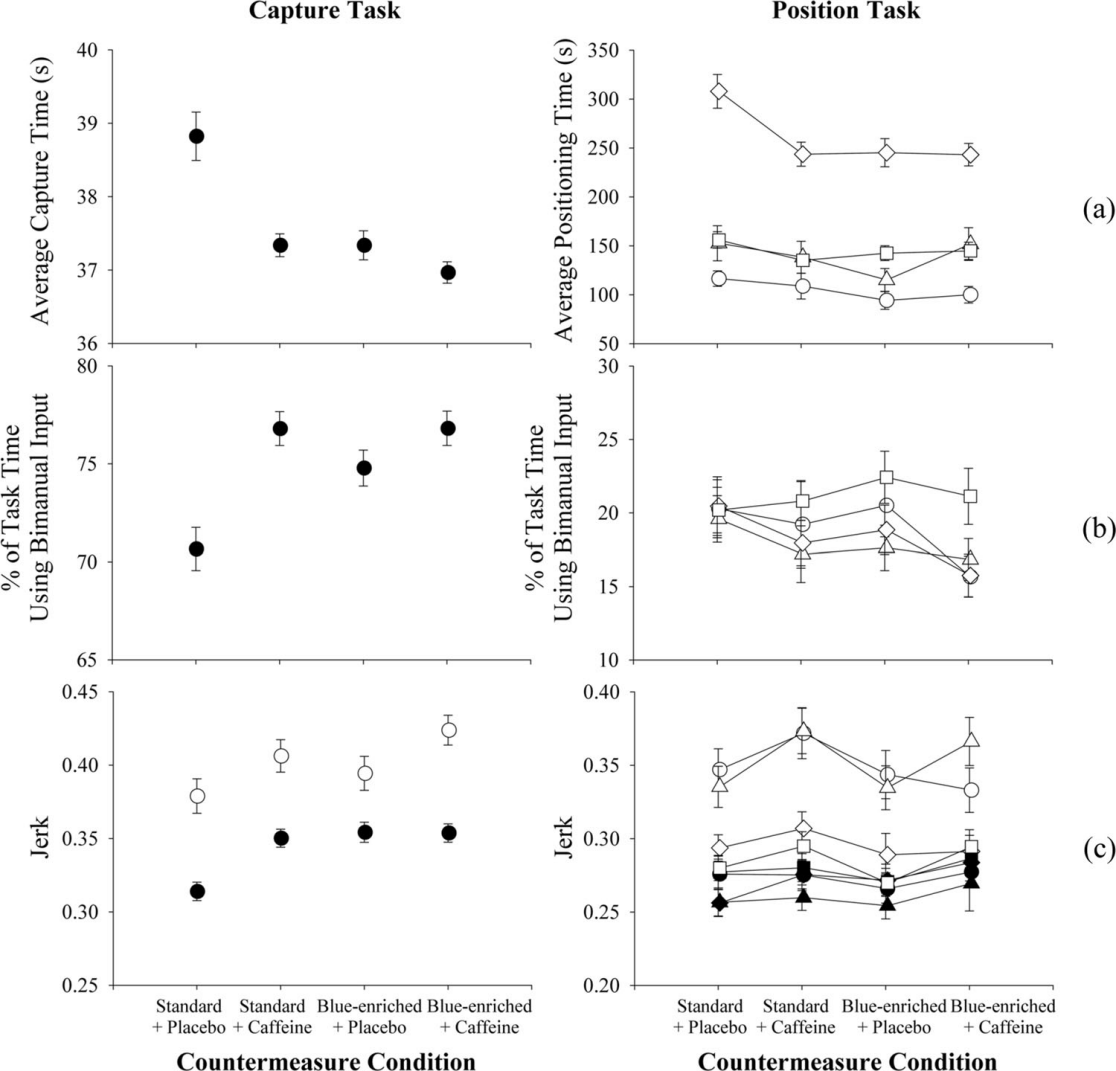
Primary task performance metrics. Error bars represent the standard deviation. Left column: Capture Task data. Right column: Position Task data. Position Task data should only be compared across countermeasure condition since the tasks in each repetition of the robotics test battery within a countermeasure condition were not comparable. Each row represents one metric: (**a**) Average completion time for one task phase, (**b**), Percentage of total time using Bimanual control, and (**c**), average Jerk for Rotation (open) and Translation (solid) Controller Inputs in the x, y, and z axes.

Figure 4 shows the effects of countermeasure conditions on the task completion time and the two metrics that characterize the control motions made by the participants during the Capture and Position Tasks: (i) percentage of time using both hands simultaneously (“bimanual control”) and (ii) jerk, which measures the inverse of smoothness of rotational controller input motions. As with the metrics discussed above, the task completion time and percentage of time using bimanual control were slightly better in the countermeasure conditions, but with no discernable difference among the three countermeasure conditions. Interestingly during the countermeasure conditions, the jerk measure was larger, indicating less smooth motions, despite the training emphasis on smooth motions.

Monitor Task data entry performance showed a similar slight improvement in all three countermeasure conditions, though none of the differences were statistically significant: (caffeine: 57.4 s ± 14.7; blue light: 60.4 s ± 18.0; both: 58.8 s ± 17.3) compared with the no countermeasure condition (68.2 s ± 17.9). The detection of clearance violations, a complex perceptual and cognitive task, did suggest a possible effect of countermeasure type, however. Caffeine use was associated with a lower average number of trials with a missed clearance violation detection (caffeine-only, 1.1 ± 1.5; both, 1.3 ± 1.5) compared to the other two conditions (none, 1.8 ± 1.1; blue-enriched light, 1.6 ± 1.7). It was also associated with a higher average number of trials in which the participant incorrectly applied the brake when no clearance violation was present (caffeine-only, 1.6 ± 1.4; both, 2.0 ± 1.7) compared to the other two conditions (none, 1.4 ± 1.7; blue-enriched light, 1.1 ± 1.4). This may indicate that participants prioritize the trade-off between task completion speed and task accuracy trade-off differently depending on their state of alertness.

## Discussion

Administration of low-dose, regularly timed caffeine was able to counteract the alertness and performance decrements in cognitive performance induced by chronic sleep loss and circadian misalignment during a simulated ISS mission. In contrast, the performance with only blue-enriched light was similar to performance with no countermeasure. These results were similar with the observed effect size from the standard cognitive and EEG measurements analyzed in Flynn-Evans et al.^1^.

We hypothesized that when performing well-practiced complex sensorimotor tasks after an episode of sleep restriction, the primary benefit of caffeine and blue-enriched white light countermeasures would be to increase the availability of visual attention resources for the operator. Since the task demands remain constant once the operator is trained, the higher reported workload is arguably due to reduced visual attention resource availability. By restoring some of this attentional capacity, caffeine or blue-light countermeasures may improve the margin for safe operations should the workload demands increase (e.g., due to lighting changes or non-normal events) or should the operator need to perform concurrent tasks. Other investigators have indeed reported that workload is more sensitive to sleep deprivation than actual performance. For example, Tomasko et al. found that well-rested participants had lower subjective workload compared to sleep-deprived participants while their performance of surgical tasks remained the same^10^.

Occasionally, some participants who completed the task before the test duration had to be prompted to begin the following test, suggesting they may have fallen asleep briefly between tests. They may have felt drowsy, but the data indicate that they usually had sufficient attention resources to maintain satisficing primary task performance during the short 1.5-to-10-min tests, resulting in a ceiling effect on primary task performance metrics. If a ceiling effect was present, one would expect the effect of countermeasure should only be evident in the secondary task measures. Since the no-countermeasure condition was always performed first in the session sequence, we are not able determine whether the maintenance of primary task performance was due to the countermeasures compensating for a reduction in attention resources due to sleep restriction and circadian shifting or whether the participants were sufficiently trained so their attentional resource utilization always remained less than what was available. In this respect, our results are different from prior studies of caffeine and blue-light countermeasures on primary task performance of driving. In nighttime driving scenarios lasting from 30–120 min, both caffeine^11–15^ and bright blue light (20 lux, 468 nm)^16^ had a beneficial effect with drivers exhibiting fewer major errors such as total lane departures. Caffeine also reduced lane deviations and steering wheel variability^13^ but blue light did not have the same effect^16^. Dim blue light given during shorter 20-min nighttime driving sessions led to similar effects to what we observed - an improvement in alertness as measured by PVT, EEG, and slow eye movements but no significant change in lane and speed deviations. Flynn-Evans et al.^1^ showed that over a 45-min period of manual driving, indications of sleepiness (i.e., the appearance of slow rolling eye movements) in their sleep-deficient subjects rose to ~20% after 15 min and ~60% after 30 min^17^. Therefore, it is possible that the effects of countermeasures observed in the extended driving experiments would also be observed during longer robotic operations.

The secondary visual attention task itself could have affected the alertness of the operator by providing additional workload that might have helped the operator avoid complacency or the under-mobilization of effort that can occur in very low workload tasks^18^. Flynn-Evans et al.^17^ observed that drivers monitoring a self-driving vehicle exhibited objective sleepiness earlier than when manually controlling their vehicle. Baulk et al.^19^ noted that automobile drivers reported less subjective sleepiness during driving trials with a secondary task and had fewer lane crossing incidents in general and EEG recordings indicating higher arousal. Gershon et al.^20^ also observed less subjective sleepiness but did not observe significant changes in lane and speed deviations. As the secondary task was present during all trials of our experiment, however, any additional alerting effect would arguably have been equivalent under all conditions for a given task.

Previous studies have shown significant individual differences in the magnitude and extent of neurobehavioral performance degradation resulting from sleep deprivation and restriction^21–23^. Individual subject performance of the primary and secondary tasks during the Capture Task trials are shown in Supplemental Fig. 1 and suggest three populations of subjects might be present. One group (Subjects 3, 13, and 15) seemed to be the most sensitive to the effects of sleep restriction and caffeine, as their performance on both the primary and secondary tasks within a session declined over time when not given caffeine. A second group (Subjects 2, 4, and 11) performed consistently well in the secondary task regardless of the countermeasure condition, mirroring the results observed by Whitney et al^23^. However, Subjects 2 and 4 showed small decreases in primary task performance over the duration of the Blue-enriched light condition, which could indicate a re-allocation of mental resources rather than true resilience. For the remaining subjects, they were able to maintain consistent primary task performance over the duration of the session, but at the expense of greater mental effort suggested by the declining secondary task performance. Within this group, the subjects showed a range of degradation from 0.5–1.5 s on the average secondary task performance. Overall, our results show similar trends among the subject group but also that there is a notable range in the effects of sleep restriction and countermeasures across these subjects.

Individual resilience to the effects of sleep restriction could be a potential crew selection criterion, however, individual resilience is also task dependent. Sleep-deprived subjects that could maintain vigilant attention during some tasks but could not perform as well for tasks requiring flexible shifting of attention control^23^. Complex tasks such as our simulated robotic operations require multiple types of attentional resources at different times during the task, so it seems unlikely that predictions based on an individual attention test would apply equally well to diverse situations.

Our experiment design of having all subjects perform the no-countermeasure condition first was primarily driven by the available resources to complete the study. This design minimized the number of randomizations that would be required for the remaining conditions and allowed us to study both types of countermeasures, which are available and commonly used during spaceflight. While the design introduces a confound in the interpretation of some of the findings, we felt that this design would provide the most conservative placement of the baseline condition. Specifically, the participants should have become progressively more sleep-deprived over the course of the study. As a result, we expect that this accumulated sleep loss would have led to poorer performance during each of the other test runs.

Analysis of both robotics and cognitive data was restricted to tests conducted during the biological night, defined as those occurring after plasma Dim Light Melatonin Onset (DLMO) time, measured for each individual prior to each condition. Tests prior to DLMO fall in the Wake Maintenance Zone (WMZ) which is a ~3-h window of reduced sleep propensity and improved neurobehavioral performance^24^. Applying this exclusion criteria affected only 6 subjects (Subjects 2, 4, 6, 10, 11, and 12) and removed 143 trials out of 4096 total trials (<3.5%). The excluded trials only occurred in the first block of the 3^rd^ session or the first two blocks of the 4^th^ experiment session as the individual’s circadian cycle shifted later due to the sleep restrictions and shifting schedule. Performance of the primary and secondary tasks during these pre-DLMO Capture Task trials did not show any consistent improvement. (Supplemental Fig. 1).

Our hypothesis that the use of caffeine and blue-enriched white light would be effective countermeasures for mitigating the effects of sleep restriction and extended time awake on robotic arm operator’s performance is partly supported by our experimental results. All the participants exhibited improved secondary visual task response time with the application of caffeine alone or together with blue-enriched light while maintaining a similar level of task performance in the primary robotic task. The cognitive, EEG, and robotics measures of alertness showed similar results with caffeine and blue-enriched light ranked based on effect size as the best countermeasure (see Table 1 in ref. ^1^). This result supports the view that results of simple cognitive tests are concordant with the effect of countermeasures on proxies of visual attention/mental workload. In practical terms, these countermeasures improve the margin of safety during real-world operations if task workload increases due to degradations in operating conditions (e.g., poor lighting) or if operators perform novel tasks which have higher attention workload compared to when they become proficient at the same task. These are both possible occurrences during robotic operations on future long-duration space missions beyond Low Earth Orbit (LEO). Further experiments are needed to determine whether the countermeasures played a role in the maintenance of primary task performance.

## Methods

**Fig. 5:**
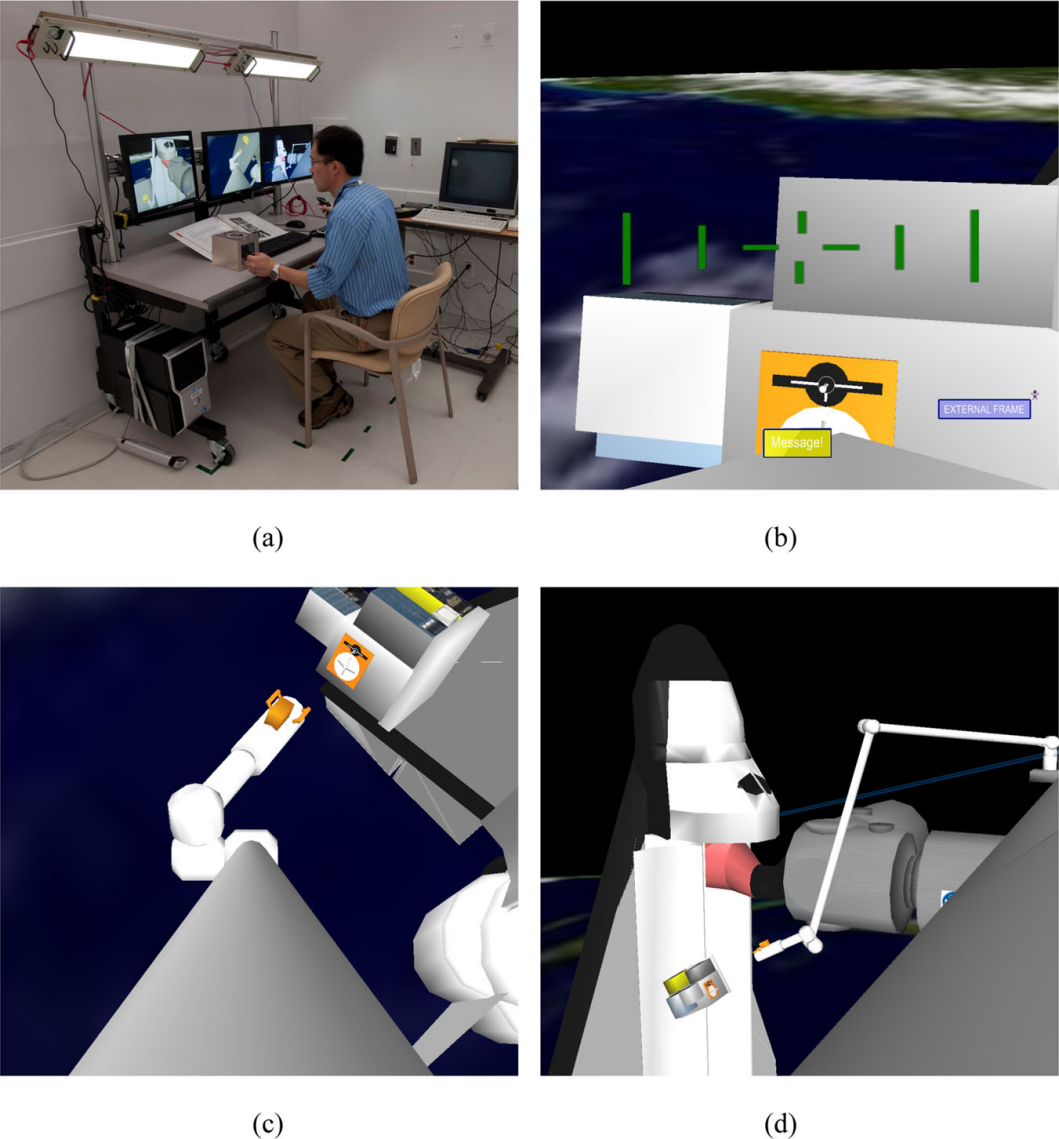
Experiment hardware configuration. **a** The configuration of the MIT RWSS and Solid State Light Modules – Research (SSLM-R) in the patient suite, which mimics their configuration on the ISS. Two additional SSLM-Rs are deployed behind the principal author (seated) at the same height and orientation as the light modules seen in the picture. During the experiment trials, the ceiling lights turned off. **b** Simulated end-effector camera view during the Position Task. The yellow box is the stimulus for the Secondary Visual Attention Task. **c** Simulated elbow camera view. **d** Simulated truss camera view. (Photos: Andrew Liu. The image in Fig. 5a has been published with permission from Andrew Liu, the principal author).

Written informed consent was obtained from all participants, and research procedures were approved by the Institutional Review Board at BWH and complied with HIPAA regulations and the Declaration of Helsinki. A detailed description of the participants and overall protocol is provided in the companion paper^1^. Here we provide details about the robotics simulation equipment and tasks. The Fig. 1 image from NASA has been published with permission from the pictured astronauts. The image in Fig. 5a has been published with permission from the first author who is shown in the image.

### MIT robotic workstation simulator

The MIT Robotics Workstation Simulator (RWSS) was developed with the Vizard Integrated Development Environment (WorldViz, Santa Barbara, CA) and has been used in several previous experiments^3,25–27^. The MIT RWSS replicates the functionality of the NASA Dynamic Skills Trainer, an astronaut training system for learning Space Station Remote Manipulator System (SSRMS) operations. The simulator interface has three monitors to provide simulated camera views, a rotational hand controller operated with the right hand, and a translational hand controller operated with the left hand (Fig. 5a). Participants can change system configurations such as camera views or arm parameters through a keyboard and mouse interface. The MIT RWSS does not model the dynamic properties of the arm and the maximum arm movement rates are much faster than in the actual system (50 cm s^−1^ vs*.* 37 cm s^−1^ max speeds in translation) to simplify and speed operations. Finally, in these experiments, participants worked alone to control the simulated arm, whereas typically, a primary and a secondary arm operator work together. The primary operator manipulates the controllers while the secondary operator assists with adjusting cameras, monitoring arm clearance and task progress.

### Simulated robotics tasks

Participants completed four repetitions of the one-hour robotics test battery within each 6-h test session (Fig. 2). The battery consisted of 12 Capture Tasks, 2 Position Tasks, and 2 Monitor Tasks that represent three classes of tasks typically executed in orbit. All participants completed the same order of tasks in each test session. The Capture Task simulates the capture of a free-flying vehicle, in this case, the H-II Transfer Vehicle (HTV). Attention is focused primarily on one camera view, although operators must check the other views to determine target spacecraft motion relative to the robot arm (Fig. 5c, d). Each trial begins with the target module and robot arm in the same initial location. Once the participant releases the robot arm brake, the target module begins to drift and rotate with a pre-determined direction and velocity. Using both hand controllers, the participant moves the arm end-effector into a pre-defined area above a grappling fixture before pulling the joystick trigger to capture the target. Participants must move efficiently to capture the target before it drifts out of range and avoid colliding with any part of the target. Each Capture task trial lasted for 90 s but participants typically completed the task within 60 s and waited the remaining time until the next trial began. Twelve unique Capture Task trials were performed at the beginning of each one-hour block of robotics testing which took a total of 20 min. The order of the 12 trials was different for each of the four test repetitions of the robotics test battery.

The Position Task simulated grappling a stationary payload and moving it to a berthing position on the ISS. Each Position Task trial had three distinctive stages. In Stage 1, Alignment, operators moved the arm from its start position to a perpendicular orientation 2 meters away (judged visually) from a grappling fixture on the target payload. Stage 1 completion time began with the participant’s initial arm movement and ended when they begin to reconfigure the arm for Stage 2. In Stage 2, Grapple, the participant first changed arm motion parameters and at least one camera view as specified by the task procedures. Then they released the arm brake and commenced the grappling task. Stage 2 completion time was measured from the start of the configuration changes to the time when the payload was successfully grappled. In Stage 3, Berth, arm parameters and camera views were changed again according to the procedures. Participants then moved the payload to a position described in the procedures. When they judged the payload to be within 2 meters and 10 degrees of the proscribed position, they pressed the ‘D’ key on the keyboard to indicate they were done with the task. Stage 3 completion time was measured from the first configuration change to the final key press. Stage 3 was similar to Stage 1 except that clearance monitoring required more attention due to the irregular shape of the payload.

The trial would automatically quit after 10 min and begin the next trial of the Robot Battery. If they finished the Position Task trial in less than 10 min, they remained seated and waited for the next trial to automatically start. Each participant completed 2 trials during each repetition of the robotics test battery for a total of 8 Position tasks during one test session. Each Position Task trial was a unique scenario (e.g., performed at different locations and with different payloads) but the two Position Task trials performed during one repetition of the robotics test battery were similar except performed on opposite sides of the ISS.

The 10-min Monitor Task simulated the automatic movement of the robotic arm from one location to another. First, the participant entered the parameters for one of two modes of motion control, Frame of Reference (FOR) mode or Joint Angle mode. For the FOR mode, they selected one of 4 parameter options from a drop-down menu, whereas in Joint Angle mode, participants manually entered the 6 joint angles of the destination as listed in the task procedures. After loading the parameters, they released the brake to start arm motion and began monitoring the automatic arm motion for clearance violations (i.e., arm motion within 1.5 meters of a structure). If they suspected a violation, participants pressed the ‘b’ key on the keyboard to stop the arm motion. A dialog box appeared acknowledging the brake application and arm motion would resume once the participant dismissed the dialog box. No feedback about the accuracy of their action was provided.

Participants completed 2 Monitor Task trials per repetition of the robotics test battery for a total of 8 trials during one test session. Each of the 8 trials was a unique scenario involving a different combination of number (0–3 violations) and location of clearance violations.

### Secondary visual attention task

During each robotics test battery trial, the participants concurrently performed a Secondary Visual Attention Task. Participants responded to the appearance of an on-screen message box alternately flashing green and yellow on the bottom of the left monitor by pressing a side button on the rotational hand controller (Fig. 5b). If they failed to respond after 10 s, the message box disappeared, and a missed response was recorded. The inter-stimulus interval was uniformly distributed between 2–10 s so approximately 4 stimuli appeared during a Capture Task trial, 30–35 stimuli during a Position Task trial, and 70–75 stimuli during a Monitor Task trial. They were instructed to give priority to the primary robotics task and forego the secondary task as necessary. As the robotics and secondary tasks both utilize visual attention, any increase in Visual Attention Task response time should reflect the prioritization of attention resources toward the robotics task.

### Participant training

Training was accomplished through a combination of PowerPoint tutorials, hands-on practice, and trainer guidance. Tutorials were self-paced and introduced basic operation concepts, terminology, and then each task type in detail. After introducing a new task, several practice trials were completed to consolidate their task strategies. Participants could ask questions at any time and repeat the practice trials as needed based on the trainer’s judgment of their progress. The amount of help and feedback from the trainer gradually decreased as training progressed. Quantitative feedback (e.g., time to compete the task, % time using multi-axis control, number of clearance violations, and number of collisions) was provided after each trial for self-assessment and to help shape any strategic or sensorimotor changes to improve performance.

Participants completed 3 training sessions each lasting 3–3.5 h, varying slightly by the participant’s rate of progress. After the first training session, a participant’s robotics aptitude was assessed by the trainer based on their performance of 12 Position Task trials. Thirteen participants failed to complete the minimum required number of trials (8 trials) with proper hand controller technique and minimal help from the instructor, thus were excused from the study. In addition, eight other participants dropped out of the study after having successfully completed at least 1 robotics training session. The Secondary Visual Attention Task was introduced into the Capture and Position task practice sessions at the end of the third training session.

On the day following their admission to the test suite, participants participated in one final review/training session. They first reviewed the PowerPoint slides to refresh their memory of the procedures and key operational rules then completed two repetitions of the robotics test battery under normal room light conditions. The order of tasks and trials was the same as the first two repetitions of the robotics test battery performed for the overnight experiment sessions.

### Data analysis and statistical methods

The jerk metric *JM* for translation and rotation were defined by Eq. 1:

1 $${\rm{JM}}=\frac{\left|{\bf{jerk}}\right|}{\left|{\bf{velocity}}\right|}$$

where **velocity** and **jerk** were calculated as the first and third (approximate) derivatives, respectively, of the translation or rotation resultant vector. As in other control movement studies, the jerk magnitude was divided by the velocity magnitude (or speed) so that the metric of jerkiness was not confounded by changes in overall movement speed^28,29^.

For the regression analysis of robotics data, separate models were created for each type of robotic task as well as each dependent variable. The countermeasure condition (Standard+Placebo, Standard+Caffeine, Blue-enriched + Placebo, Blue-enriched+Caffeine), Robotics Battery repetition (1–4), and overnight session order (3 orders) were the independent fixed effect variables and the subject was included as a random effect. To meet Fisher’s criteria, the Secondary Visual Attention Task response time data were inverse transformed. The final models for each outcome yielded normally distributed residuals, confirmed by the Kolmogorov-Smirnov test of normality and the Levene’s test of homoscedasticity. For the number of secondary task stimuli presented in a trial, the data for the Capture Task was square root transformed. Since the task completion time also affected the number of stimuli shown, the regression model included task completion time as a co-variate. Using a metric normalized over the maximum time allotted to each trial (90 s) did not lead to a regression model that yielded homoscedastic residuals. In analyzing the data from the Position Task trials, the number of secondary task stimuli presented was normalized to a 10-min baseline to account for variable task completion time. The data from the Monitor Task trials was square root transformed. The regression model for the Monitor Task data was normally distributed but not homoscedastic.

For the primary robotic performance measures, regression models could not be fit such that the residuals met the assumptions of normality and homoscedasticity. Instead, a Kruskal-Wallis test was used to determine whether there were any significant differences between the countermeasure groups. If the test returned a significant result, a series of Conover-Iman pairwise comparisons were made to determine differences between the countermeasure conditions.

### Reporting summary

Further information on research design is available in the Nature Research Reporting Summary linked to this article.

## Data availabilty

De-identified individual data for all outcomes described are available upon request from the corresponding author, subject to appropriate ethical approval and completion of a Data Sharing Agreement. Data will be provided immediately upon completion of these conditions.

### Supplementary information

Reporting Summary

Supplemental Material
