# Supplementary material for: Effects of caffeine and blue-enriched light on spare visual attention during simulated space teleoperation
Source: NPJ Microgravity. 2023 Dec 19;9:94. doi: 10.1038/s41526-023-00299-8 (PMC10730832; doi:10.1038/s41526-023-00299-8)
Supplement: Supplementary file 1 — Reporting Summary [file 41526_2023_299_MOESM1_ESM.pdf]

nature portfolio

Corresponding author(s): Andrew Liu

Last updated by author(s): Dec 27, 2022

Reporting Summary

Nature Portfolio wishes to improve the reproducibility of the work that we publish. This form provides structure for consistency and transparency in reporting. For further information on Nature Portfolio policies, see our Editorial Policies and the Editorial Policy Checklist.

Statistics

For all statistical analyses, confirm that the following items are present in the figure legend, table legend, main text, or Methods section.

n/a	Confirmed	
☐	☒	The exact sample size (n) for each experimental group/condition, given as a discrete number and unit of measurement
☒	☐	A statement on whether measurements were taken from distinct samples or whether the same sample was measured repeatedly
☐	☒	The statistical test(s) used AND whether they are one- or two-sided<br>*Only common tests should be described solely by name; describe more complex techniques in the Methods section.*
☐	☒	A description of all covariates tested
☐	☒	A description of any assumptions or corrections, such as tests of normality and adjustment for multiple comparisons
☐	☒	A full description of the statistical parameters including central tendency (e.g. means) or other basic estimates (e.g. regression coefficient) AND variation (e.g. standard deviation) or associated estimates of uncertainty (e.g. confidence intervals)
☐	☒	For null hypothesis testing, the test statistic (e.g. F, t, r) with confidence intervals, effect sizes, degrees of freedom and P value noted<br>*Give P values as exact values whenever suitable.*
☒	☐	For Bayesian analysis, information on the choice of priors and Markov chain Monte Carlo settings
☐	☒	For hierarchical and complex designs, identification of the appropriate level for tests and full reporting of outcomes
☐	☐	Estimates of effect sizes (e.g. Cohen's d, Pearson's r), indicating how they were calculated

Our web collection on statistics for biologists contains articles on many of the points above.

Software and code

Policy information about availability of computer code

Data collection	The MIT Robotics Workstation Simulator (RWSS) was developed with the Vizard Integrated Development Environment, version 5 (WorldViz, Santa Barbara, CA) and replicates the functionality of the NASA Dynamic Skills Trainer, an astronaut training system for learning Space Station Remote Manipulator System (SSRMS) operations. The simulation scripts are written in Python and the 3-D models were created using 3D Studio Max, Blendr, or AC3D.
Data analysis	Data were analyzed using SYSTAT and MATLAB, using the tests within these software tools as described in the manuscript. MATLAB m-files were created to perform basic statistics and the plots.

For manuscripts utilizing custom algorithms or software that are central to the research but not yet described in published literature, software must be made available to editors and reviewers. We strongly encourage code deposition in a community repository (e.g. GitHub). See the Nature Portfolio guidelines for submitting code & software for further information.

Data

Policy information about availability of data

All manuscripts must include a data availability statement. This statement should provide the following information, where applicable:

- Accession codes, unique identifiers, or web links for publicly available datasets
- A description of any restrictions on data availability
- For clinical datasets or third party data, please ensure that the statement adheres to our policy

De-identified individual data for all outcomes described are available upon request from the corresponding author, subject to appropriate ethical approval and completion of a Data Sharing Agreement. Data will be provided immediately upon completion of these conditions.

Human research participants

Policy information about studies involving human research participants and Sex and Gender in Research.

Reporting on sex and gender	We collected data from 17 participants, of whom 7 were female and 10 were male. This is reported in the manuscript. We did not conduct sex specific analyses because we did not have funding to collect data from enough individuals to meet power requirements for stratified analyses. We did consider sex as a potential covariate in our initial analyses, but it was not a significant factor and was therefore removed from analysis.
Population characteristics	Our participants were healthy research volunteers (n = 17, 7F, mean age ± SD: 37.1 ± 8.1 years), aged 26-55 years.
Recruitment	Participants were recruited through advertisements to the general public. These advertisements were IRB approved and physical flyers were placed in cafes and stores. Digital versions of these flyers were placed in online community forums (e.g., Craig's list and Facebook). Interested participants contacted recruiting staff and completed an initial phone screen to determine eligibility. Candidates who passed the phone screen were invited to attend a comprehensive screening that included a medical and psychological examination and questionnaire-based screening. We have no reason to suspect that our recruiting methods were biased towards or against any group.
Ethics oversight	MIT COUHES has accepted the decision of the Brigham and Women's IRB to approve the protocol.

Note that full information on the approval of the study protocol must also be provided in the manuscript.

Field-specific reporting

Please select the one below that is the best fit for your research. If you are not sure, read the appropriate sections before making your selection.

☐ Life sciences ☒ Behavioural & social sciences ☐ Ecological, evolutionary & environmental sciences

For a reference copy of the document with all sections, see nature.com/documents/nr-reporting-summary-flat.pdf

Behavioural & social sciences study design

All studies must disclose on these points even when the disclosure is negative.

Study description	Healthy research volunteers maintained a regular sleep-wake schedule at home, with six hours time in bed per night for one week prior to a 13-day inpatient study at the Intensive Physiologic Monitoring Unit (IPM), Brigham and Women's Hospital. The inpatient study is described in detail in the manuscript, along with a protocol schematic. In brief, participants completed two robotics training sessions upon admission to the laboratory. They next completed four cycles of 'slam shifting' by which their sleep-wake cycle was inverted. During the first slam shift, they completed a six-hour robotics test session and during the second 'day' of the slam shift, they were kept in dim light for assessment of the dim light melatonin onset. The first slam shift involved a baseline condition with no countermeasures. The following three slam shift blocks were randomized between three countermeasures, including caffeine and blue-enriched light, placebo and blue-enriched light, and caffeine and standard light.
Research sample	The participants were recruited from the general population, but they were required to meet criteria consistent with the NASA astronaut population, including: age between 26-59, a college degree or higher, good physical health, which was assessed by medical history, physical examination, blood biochemistry and hematology, and electrocardiogram, good mental health, which was evaluated by interview with a staff psychologist/psychiatrist, normal sight, including the absence of color-blindness, which was confirmed by an ophthalmologic examination before and after the study and no history of sleep and circadian-rhythm disorders.
Sampling strategy	Since we were budget-limited in the number of participants we could study, we used the following approach to determine our power: to detect an effect of SLEEP_CONDITION on any measured variable with 95% confidence, the normalized squared difference between group means (X1 - X2)2/ s2 will have to be greater than the critical value F(1,22; 0.05)/6 = 4.30/6 = 0.72 = 0.852, in which s2 = the pooled variance estimate for individual measurements. That is, the difference between group means will have to be more than

85% of the pooled estimate, s, of the standard deviation in order to be detected with that confidence.

Data collection	Each 6-hour test session consisted of four repetitions of a one-hour robotics test block followed by a 30-minute cognitive test block. The 1-hour robotics test block consisted of 10 Capture ("Track & Capture") tasks, 2 Position (“Fly-to and Grapple”) tasks, and 2 Monitor ("Autosequence") tasks and were presented in the same order in each block<br><br>The Capture Task simulates the capture of a free-flying vehicle, in this case, the H-II Transfer Vehicle (HTV). Attention is focused primarily on one camera view, although operators must check the other views to determine target spacecraft motion relative to the robot arm. Each trial begins with the target module and robot arm in the same initial location. Once the participant releases the robot arm brake, the target module begins to drift and rotate with a pre-determined direction and velocity. Using both hand controllers, the participant moves the arm end-effector into a pre-defined area above a grappling fixture before pulling the joystick trigger to capture the target. Participants must move efficiently to capture the target before it drifts out of range and avoid colliding with any part of the target. Each Capture task trial lasted for 90 seconds but participants typically completed the task within 60 seconds and waited the remaining time until the next trial began. Twelve unique Capture Task trials were performed at the beginning of each one-hour block of robotics testing which took a total of 20 minutes. The order of the 12 trials was different for each of the four test repetitions of the robotics test battery.<br><br>The Position Task simulated grappling a stationary payload and moving it to a berthing position on the ISS. Each Position Task trial had distinctive three stages. In Stage 1, Alignment, operators moved the arm from its start position to a perpendicular orientation 2 meters away (judged visually) from a grappling fixture on the target payload. Stage 1 completion time began with the participant’s initial arm movement and ended when they begin to reconfigure the arm for Stage 2. In Stage 2, Grapple, the participant first changed arm motion parameters and at least one camera view as specified by the task procedures. Then they released the arm brake and commenced the grappling task. Stage 2 completion time was measured from the start of the configuration changes to the time when the payload was successfully grappled. In Stage 3, Berth, arm parameters and camera views were changed again according to the procedures. Participants then moved the payload to a position described in the procedures. When they judged the payload to be within 2 meters and 10 degrees of the proscribed position, they pressed the ‘D’ key on the keyboard to indicate they were done with the task. Stage 3 completion time was measured from the first configuration change to the final key press. Stage 3 was similar to Stage 1 except that clearance monitoring required more attention due to the irregular shape of the payload. A trial would automatically quit after 10 minutes and begin the next trial of the Robot Battery. If they finished the Position Task trial in less than 10 minutes, they remained seated and waited for the next trial to automatically start. Each participant completed 2 trials during each repetition of the robotics test battery for a total of 8 Position tasks during one test session. Each Position Task trial was a unique scenario (e.g., performed at different locations and with different payloads) but the two Position Task trials performed during one repetition of the robotics test battery were similar except performed on opposite sides of the ISS.<br><br>The 10-minute Monitor Task simulated the automatic movement of the robotic arm from one location to another. First, the participant entered the parameters for one of two modes of motion control, Frame of Reference (FOR) mode or Joint Angle mode. For the FOR mode, they selected one of 4 parameter options from a drop-down menu, whereas in Joint Angle mode, participants manually entered the 6 joint angles of the destination as listed in the task procedures. After loading the parameters, they released the brake to start arm motion and began monitoring the automatic arm motion for clearance violations (i.e., arm motion within 1.5 meters of a structure). If they suspected a violation, participants pressed the ‘b’ key on the keyboard to stop the arm motion. A dialog box appeared acknowledging the brake application and arm motion would resume once the participant dismissed the dialog box. No feedback about the accuracy of their action was provided. Participants completed 2 Monitor Task trials per repetition of the robotics test battery for a total of 8 trials during one test session. Each of the 8 trials was a unique scenario involving a different combination of number (0-3 violations) and location of clearance violations.<br><br>The 30-minute cognitive test block included the visual analogue scale (VAS), the Karolinka Sleepiness Scale (KSS), the Digit Symbol Substitution Task (DSST), the 10-minute visual version of the Psychomotor Vigilance Test (PVT), and the Karolinska Drowsiness Test (KDT). Each of these tests was administered on a standardized testing computer that was used solely for the purpose of administering these tests. (see details in Flynn-Evans et al., accompanying manuscript)
Timing	Each 6-hour test session (see Figure 1 in the manuscript) consisted of four repetitions of a one-hour robotics test block followed by a 30-minute cognitive test block. These testing blocks were timed to be 10 hours after each participant's wake time and occurred at the same time for each individual.
Data exclusions	We restricted our analysis to tests that occurred after an individual’s timing of DLMO in order remove the influence of the circadian wake-maintenance zone in our analyses. Less than 4% of the data were excluded.
Non-participation	One participant (3163V) had to leave the study early due to a family emergency - this is noted in the accompanying manuscript (Flynn-Evans et al.). Thirteen subjects were excluded after the first training session for not meeting baseline performance and eight others chose to drop out after completing the first robotics training session. This is stated in the manuscript.
Randomization	Participants were randomized to the countermeasure order by an experienced researcher who was not involved in the study.

Reporting for specific materials, systems and methods

We require information from authors about some types of materials, experimental systems and methods used in many studies. Here, indicate whether each material, system or method listed is relevant to your study. If you are not sure if a list item applies to your research, read the appropriate section before selecting a response.

Materials & experimental systems

n/a	Involved in the study
☒	☐ Antibodies
☒	☐ Eukaryotic cell lines
☒	☐ Palaeontology and archaeology
☒	☐ Animals and other organisms
☒	☐ Clinical data
☒	☐ Dual use research of concern

Methods

n/a	Involved in the study
☒	☐ ChIP-seq
☒	☐ Flow cytometry
☒	☐ MRI-based neuroimaging
