# Supplementary material for: Effects of caffeine and blue-enriched light on spare visual attention during simulated space teleoperation
Source: NPJ Microgravity. 2023 Dec 19;9:94. doi: 10.1038/s41526-023-00299-8 (PMC10730832; doi:10.1038/s41526-023-00299-8)
Supplement: Supplementary file 2 — Supplemental Material [file 41526_2023_299_MOESM2_ESM.pdf]

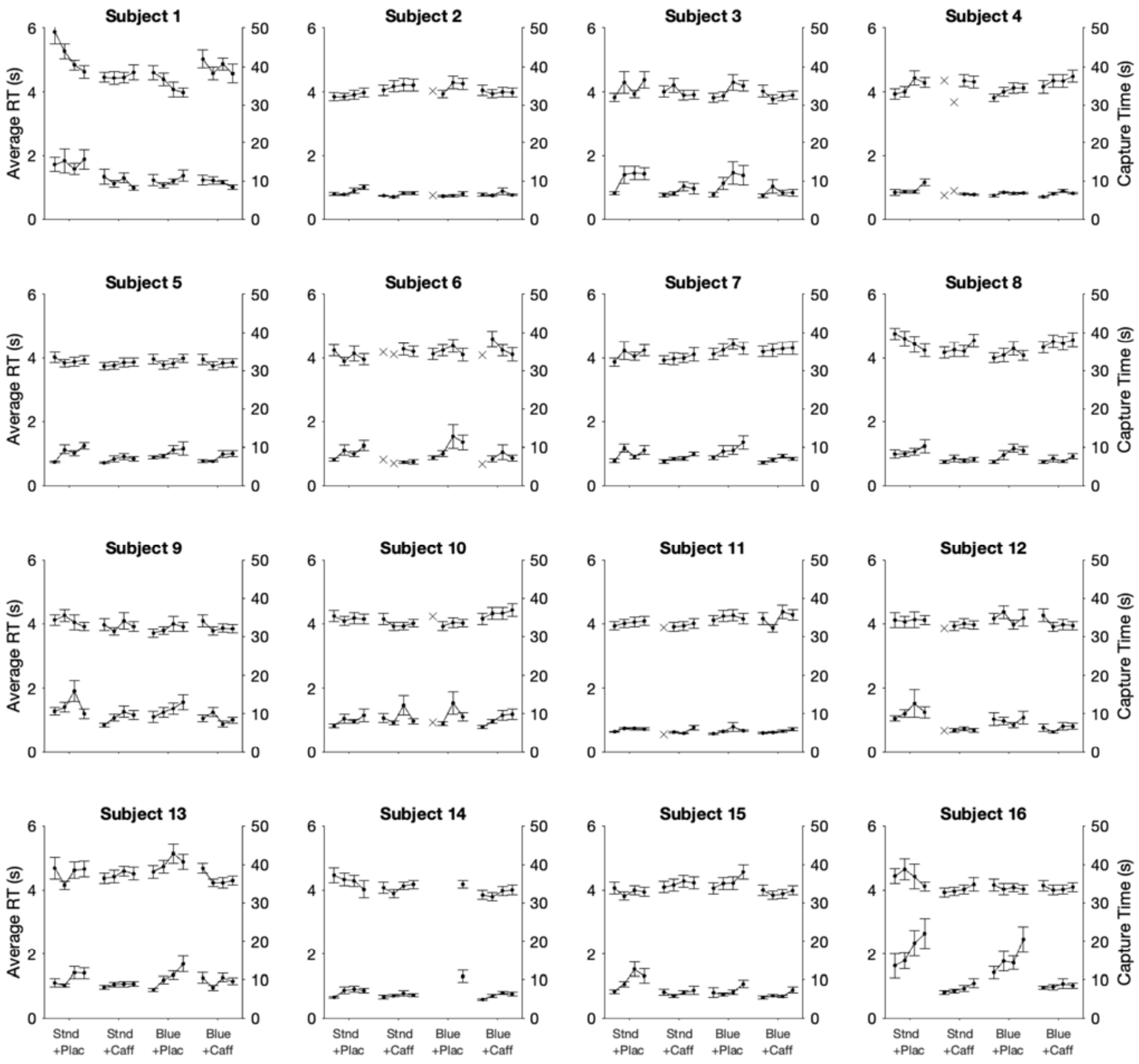

Supplemental Figure 1 – Individual subject Capture Task performance. Secondary and primary task performance plotted by individual subject. The average response time during the secondary task (lower data points and left y-axis) and average completion time of the Capture Task (upper data points and right y-axis) are shown together. The X symbol for Subjects 2, 4, 6, 10, 11, and 12 indicate performance in trials occurring before DLMO. These trials were excluded from the analysis due to possible effects that can occur in the Wake Maintenance Zone.
